# Parenting Resilient Kids (PaRK), an online parenting program to prevent anxiety and depression problems in primary school-aged children: Study protocol for a randomised controlled trial

**DOI:** 10.1186/s13063-018-2605-8

**Published:** 2018-04-19

**Authors:** Luwishennadige Madhawee N. Fernando, Wan Hua Sim, Anthony F. Jorm, Ron Rapee, Katherine A. Lawrence, Marie B. H. Yap

**Affiliations:** 1 Monash Institute of Cognitive and Clinical Neurosciences, Monash University, Clayton, VIC Australia; 2 Melbourne School of Population and Global Health, University of Melbourne, Melbourne, VIC Australia; 3 Centre for Emotional Health, Department of Psychology, Macquarie University, Sydney, NSW Australia

**Keywords:** Prevention, Mental health, Universal, Internalising, Internet, Childhood

## Abstract

**Background:**

Preventive efforts targeting childhood anxiety and depression symptoms have the potential to alter the developmental trajectory of depression and anxiety disorders across the lifespan. Substantial previous research suggests that modifiable parenting factors such as parental aversiveness and over-involvement are associated with childhood anxiety, depressive and internalising symptoms, indicating that parents can play a critical role in prevention. The Parenting Resilient Kids study is a new evidence-based online parenting program designed to prevent anxiety and depression problems in primary school-aged children by reducing family-based risk factors and enhancing protective factors through increased positive interactions between parent and child.

**Methods/design:**

The current study is a parallel group superiority randomised controlled trial with parent-child dyads randomised to the intervention or active-control group in a 1:1 ratio. The intervention group will receive the Parenting Resilient Kids program consisting of a feedback report on parenting behaviours and up to 12 interactive online modules personalised based on responses to the parent survey. The active-control group will receive a standardised package of online educational materials about child development and wellbeing. The trial website is programmed to run a stratified random allocation sequence (based on parent gender) to determine group membership. We aim to recruit 340 parent-child dyads (170 dyads per group). We hypothesise that the intervention group will show greater improvement in parenting risk and protective factors from baseline to 3-month follow-up (primary outcome), which will in turn mediate changes in child depressive and anxiety symptoms from baseline to 12 and 24 months (co-primary outcomes). We also hypothesise that the intervention group will show greater benefits from baseline to 3-, 12- and 24-month follow-up, with regard to: child depressive and anxiety symptoms (co-primary outcomes); and child and parent health-related quality of life, and overall family functioning (secondary outcomes).

**Discussion:**

This randomised controlled trial will examine the efficacy of the Parenting Resilient Kids program as a preventive intervention for anxiety and depression symptoms in primary school-aged children, as well as changes in child and parent health-related quality of life. Findings from this study will examine design features that render web-based prevention programs effective and the extent to which parents can be engaged and motivated to change through a minimally guided parenting program.

**Trial registration:**

Australian New Zealand Clinical Trials Registry (ANZCTR): Trial ID ACTRN12616000621415 Registered on 13 May 2016. Updated on 3 March 2017.

**Electronic supplementary material:**

The online version of this article (10.1186/s13063-018-2605-8) contains supplementary material, which is available to authorized users.

## Background

Depression and anxiety disorders tend to start early in life and give rise to a cascade of negative long-term consequences. A meta-analysis of the age of onset for anxiety disorders shows that separation anxiety and specific phobia show an age of onset from 10 to 11 years indicating that primary school years could be a significant risk phase for developing anxiety disorders [1]. Longitudinal studies have found a significant association between early-onset anxiety disorders and later risk of anxiety and depressive disorders in adulthood, nicotine, alcohol and illicit drug dependence, under-achievement in education, and early parenthood [2]. The recurrence of depressive disorders in adulthood following a childhood episode of major depressive disorder has been estimated to be as high as 75% [3]. Therefore, preventive efforts targeting childhood anxiety and depression syptoms have the potential to alter the developmental trajectory of anxiety and depressive disorders across the lifespan.

Consideration of family and parenting factors in the prevention of depression and anxiety has been recognised as a key research translation priority [4–6]. Yap and Jorm conducted a systematic review of the complex literature examining links between parental factors and depression or anxiety problems in children aged 5–11 [5]. They identified a range of modifiable parental factors, such as warmth, aversiveness and over-involvement, which were associated with childhood depressive symptoms, anxiety symptoms, and internalizing problems. They also noted that some parenting factors, namely abuse, inter-parental conflict and over-involvement, have not been adequately incorporated into existing prevention and intervention programs. Therefore, there is a clearly identified need for translation of the evidence regarding risk and protective factors into specific, practical strategies that parents can use to reduce the risk of depression and anxiety in their children.

Parenting-focused preventative interventions have been found to reduce externalising and internalising problems as well as increase positive attributes such as self-regulation and self-esteem [7]. Yap et al. conducted a meta-analysis of randomised controlled trials (RCTs) of parenting interventions to prevent internalising problems in children and reported small but significant effects in reducing child internalising (including both depression and anxiety) problems in the longer term [8]. However, Morawska and Sanders argue that the capacity for preventative parenting interventions to produce valued outcomes is often compromised by poor parental engagement [9]. While perceived practical barriers, such as difficulties with accessibility (timing, frequency, location), have been shown to lead to poor engagement, using different delivery modalities and self-directed interventions has been shown to promote engagement [9].

Web-based programs reduce geographical and time constraints and can also be anonymous, potentially increasing engagement for individuals concerned about privacy, confidentiality, and stigma. Furthermore, online programs can be disseminated widely at low cost, making universal preventive efforts feasible. A meta-analysis of 12 web-based parenting programs showed statistically significant medium effects across parent and child outcomes, suggesting that parenting interventions can be delivered effectively using online platforms [10]. There is also considerable support for effectiveness of web-based prevention and intervention programs for child and adolescent anxiety and depression [11–13]. Given the advantages of online delivery, and the success of online programs to date, the development of web-based preventive parenting interventions has been recommended as an important strategy to increase participation in preventive programs [14, 15].

## Development of ‘Parenting Resilient Kids’

The ‘Parenting Resilient Kids’ (PaRK) intervention is part of the broader Parenting Strategies program (www.parentingstrategies.net), and modelled on two other existing web-based parenting interventions [16, 17]. It was developed as a web-based preventive parenting intervention for depression and anxiety in primary school-aged children. The content of the program is derived from the Parenting Guidelines ‘How to reduce your child’s risk of depression and clinical anxiety: strategies for parents of primary school-aged children’, which will be referred to as Guidelines hereafter [18]. The Guidelines were developed based on a systematic review of parental factors associated with anxiety, depression, and internalising problems in children aged 5–11, which parents can potentially modify [5], as well as a Delphi expert consensus study to translate the evidence into practical strategies. A recent online survey evaluation of the Guidelines supports their acceptability among both parents and professionals, and their dissemination as a universal resource for the prevention of childhood depression and anxiety [19]. Parents who intend to put the Guidelines into practice may require further support in identifying individual needs as well as in building skills in the domains identified in the Guidelines. To facilitate this process, an online parenting program was developed which includes: (a) an online self-assessment parenting scale, which provides a personalised feedback report; and (b) an interactive parenting program comprising up to 12 modules. Table 1 shows the Guidelines and the corresponding subsections of the parenting scale and module components.

**Table 1:** The Guidelines and the corresponding subsections of the parenting scale and module components

Guidelines subheading	Corresponding subsection of the parenting scale and feedback report	Title of interactive module	Outline of content	Rationale for inclusion
You can reduce your child’s risk of developing depression and clinical anxiety	NA. Not included in parenting scale or feedback report.	Not included in the modules	Psychoeducation about the role of parents in the prevention of depression and anxiety in primary school-aged children.	Endorsed by experts.
Establish and maintain a good relationship with your child	Relationship with your child	Topic 1: Show affection and acceptance	Helps parents show their children physical affection and acceptance through words and actions.	Sound evidence that parental warmth is associated with less internalizing symptoms. Emerging evidence that parental warmth is associated with fewer depression symptoms.
		Topic 4: Make time to talk	Helps parents develop a supportive relationship with their child by learning effective ways to talk and listen to their child.	
Be involved and support increasing autonomy	Involvement in your child’s life	Topic 2: Be involved	Helps parents stay involved and interested in their child’s life.	Emerging evidence that parent’s knowledge regarding their child′s activities, whereabouts and friends (parental monitoring) is associated with less anxiety and depressive symptoms.
		Topic 3: Encourage autonomy	Helps parents encourage increasing age-appropriate autonomy in their child’s life.	Sound research evidence that over-involvement and autonomy granting are risk and protective factors, respectively, for depression; endorsed by experts.
Encourage supportive relationships	Child’s relationships with others	Topic 9: Topic Encourage supportive relationships	Provides strategies for parents to support their child’s social skills development.	Emerging evidence that parental encouragement of sociability is associated with less child anxiety; endorsed by experts.
Establish family rules and consequences	Rules and consequences for your child	Topic 6: Establish family rules and consequences	Highlights the importance of consistent and clear boundaries for child’s behaviours, and provides specific strategies to establish these.	Emerging evidence of the association between inconsistent discipline and internalizing symptoms; endorsed by experts.
Encourage good health habits	Health habits	Topic 5: Encourage healthy habits	Provides strategies to help parents encourage good health habits in their child, including a healthy diet, physical activity, good sleep habits, and appropriate screen time.	Endorsed by experts
Minimise conflict in the home	Home environment	Topic 10: Manage conflict in the home	Addresses the need for adaptive conflict management between parents, and between parent and child, and provides specific strategies to do these.	Evidence that inter-parental conflict and aversiveness (including parent-child conflict) are risk factors for depression (sound evidence) and anxiety (emerging evidence); endorsed by experts.
Help your child to manage emotions	Managing emotions	Topic 8: Help your child manage emotions	Helps parents understand and talk about their child’s emotions as well as provides parents strategies to help children manage strong emotions.	Emerging evidence that parents modelling anxiety is associated with anxiety symptoms in children. Endorsed by experts.
Help your child to set goals and solve problems	Setting goals and dealing with problems	Topic 7: Help set goals and solve problems	Provides strategies for parents to help their children develop good problem-solving skills.	Endorsed by experts.
Support your child when something is bothering them	Dealing with negative emotions	Topic 11: Help your child manage anxiety	Provides strategies for parents to help their children manage their everyday anxiety.	Emerging evidence that parents modelling anxiety is associated with anxiety symptoms in children. Endorsed by experts.
Help your child to manage anxiety so that it does not become a problem	Dealing with negative emotions	Topic 11: Help your child manage anxiety	Provides strategies for parents to help their children manage their everyday anxiety.	Emerging evidence that parents modelling anxiety is associated with anxiety symptoms in children. Endorsed by experts.
Encourage professional help seeking when needed	Getting help when needed	Topic 12: Seek help	Helps parents understand what depression and anxiety problems can look like in primary school- aged children and what they can do if their child is/becomes unwell.	Endorsed by experts; evidence that parents are important conduits to children seeking professional help for mental health problems.
	Do not blame yourself (not included in the Guidelines and parenting scale, but included in feedback report for all parents)	NA. No module on this topic.	Aims to dispel guilt/self-blame in parents.	Endorsed by experts.

The Parenting Resilient Kids program was also developed to fulfill many of the principles of persuasive systems design, a concept defined as “computerised software or information systems designed to reinforce, change or shape attitudes or behaviours [20]. A systematic review by Kelders and colleagues suggests that adherence to web-based interventions can be improved by incorporating persuasive technology elements into the design [21]. For example, persuasive design features of the program include tailoring (of feedback and parenting program), personalisation (the parenting program offers participants their own personalised dashboard, diary pages etc.) and self-monitoring (of progress through goals and modules).

The online parenting scale was developed to evaluate a wide range of potentially modifiable parenting practices against the 12 parenting domains covered in the Parenting Guidelines. This scale is a criterion-referenced measure that is modelled on two other published measures used in the existing Parenting Strategies interventions [22, 23]. Parents’ responses to the items in the parenting scale are used to determine the content of the individually tailored feedback report, which will be received by parents in the intervention group. To automate the personalisation of the feedback content, a system of scoring the scale responses was developed whereby algorithms were created for all possible combinations of responses on each parenting domain on the scale. The algorithms link each possible combination of scale responses to the corresponding feedback messages that are appropriate to the responses selected and the score obtained for the parenting domain. The resulting personalised feedback report highlights individual strengths and areas for improvement in the different parenting domains. Where relevant, links are provided for parents to seek additional information from external websites if they wish. The feedback messages are designed to be brief and provide an overview of the parenting modules to encourage parents seeking further information to refer to the modules. The content of the scale and feedback messages were initially drafted by a doctoral student with postgraduate qualifications in psychology (WHS) and evaluated by the research team (comprising WHS, MBHY, AFJ, KAL) to ensure their fidelity to the Guidelines.

The web-based parenting program consists of 12 modules carefully selected to encompass the content of the Guidelines. Table 1 outlines the module titles and rationale for including each module. The modules are presented in a conversational tone and are designed to be engaging, informative and interactive. Each module includes illustrations, tasks designed to help parents troubleshoot parenting challenges, vignettes, interactive tasks that provide further practice for skills learnt in the modules, goal-setting tasks and an end-of-module quiz with immediate feedback. Module content was initially drafted by a doctoral student with graduate qualifications in psychology (LMNF) and evaluated by the research team (comprising LMNF, MBHY, AFJ, and KAL) to ensure their fidelity to the Guidelines as well as to other relevant evidence and credible resources.

Target end-users (parents of primary school-aged children) were invited to co-design the program by participating in reference groups. Fourteen parents of primary school-aged children (5–11 years) were recruited via staff newsletters across Monash University and the University of Melbourne. Parents participated in a 2-hour workshop in which they reviewed the text of the parenting scale and a mock feedback report, focusing on the acceptability of the language, and the logic of the feedback messages. Parents also reviewed two modules for language, tone, degree of interactivity and density of content. Parents who had agreed to be contacted on an ad hoc basis following the workshop, were consulted regarding the design and layout of the registration website and online scales, the usefulness of feedback messages generated from the parenting scale, and as well as content and presentation of modules.

## Aim and hypotheses

This RCT will compare the effects of providing parents with the Parenting Resilient Kids program (intervention) to a standardised package of educational factsheets about child development and wellbeing.

Specifically, we hypothesise that:

- H1: The intervention group will show greater improvement than the control group in parenting risk and protective factors from baseline to 3-month follow-up (primary outcome), which will in turn mediate changes in child depressive and anxiety symptoms from baseline to 12 and 24 months (co-primary outcomes).
- H2: The intervention group will show greater benefits than the control group from baseline to 3-, 12- and 24-month follow-up, with regard to:
  - H2a. Child depressive and anxiety symptoms (co-primary outcomes); and
  - H2b. Child and parent health-related quality of life, and overall family functioning (secondary outcomes).

## Methods/design

### Trial design

**Fig. 1:**
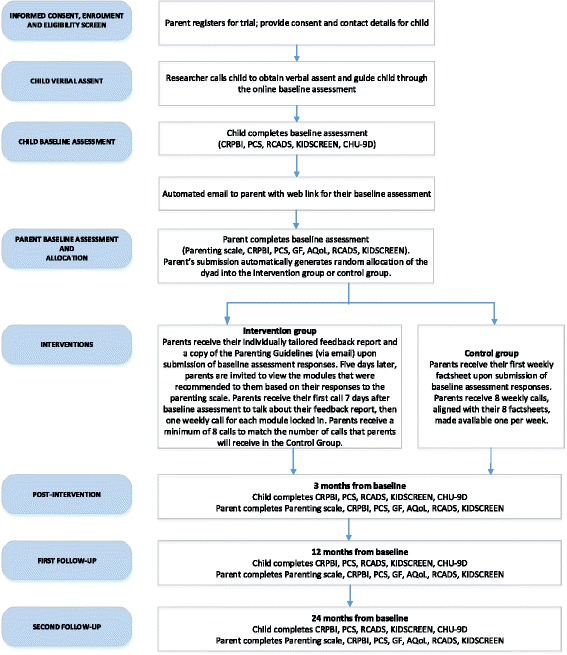
Trial protocol. Children’s Report of Parent Behaviour Inventory (CRPBI), Psychological Control Scale (PCS), RCADS (Revised Children’s Anxiety and Depression Scale), Child Health Utility (CHU-9D), Assessment of Quality of Life (AQoL-8D), General Functioning subscale of the McMaster Family Assessment Device (GF)

**Fig. 2:**
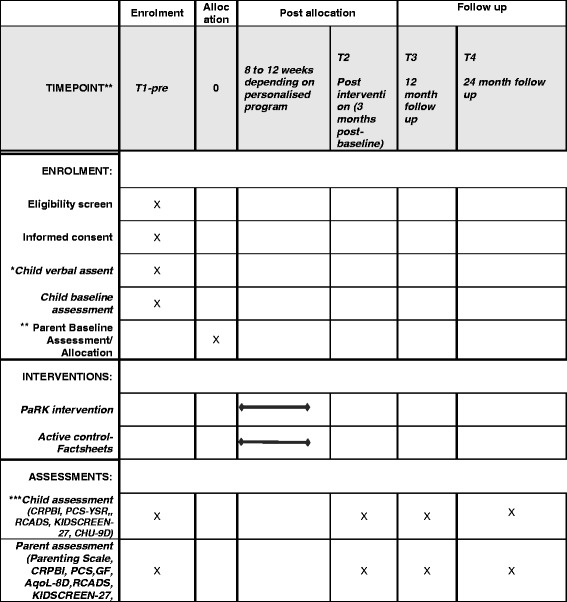
SPIRIT figure

A parallel group superiority RCT was designed with parent-child dyads randomised to the intervention or active-control group in a 1:1 ratio. The trial can be considered a ‘pilot’ to determine the efficacy of the Parenting Resilient Kids program with the view to disseminate the program more widely in the future. All participants will be assessed at baseline, post-intervention (3 months after baseline), at 9-month follow-up (12 months after baseline) and 21-month follow-up (24 months after baseline). A dedicated website has been set up to take registrations, run the program, and collect and store data. The trial protocol flow diagram is outlined in Fig. 1. Further details about the protocol are reported in the SPIRIT figure (Fig. 2) and SPIRIT checklist (Additional file 1).

### Ethical approval and trial registration

This study has been approved by the Monash University Human Research Ethics Committee (MUHREC ID CF16/152–2016000063), the Department of Education and Training (DET) Victoria, and Catholic Education Melbourne. Approvals for research study promotion and recruitment have also been received from the relevant authorities and schools in other states of Australia. The study is registered on the Australian New Zealand Clinical Trials Registry (ANZCTR) - Trial ID: ACTRN12616000621415.

### Participants

#### Sample size

Based on a power analysis for a repeated-measures design with a power of 0.80, small effect (Cohen’s d = 0.2), and alpha of 0.05 two-tailed, a sample size of 294 parent-child dyads (147 dyads per group) is required. Allowing for an attrition of approximately 10–15%, we aim to recruit 340 parent-child dyads (170 dyads per group).

#### Recruitment

Parents will be recruited using a combination of methods. The primary recruitment strategy will involve reaching parents through school networks. Primary schools across Australia will be contacted by the research team, to request for the promotional material to be disseminated via any means of parent communication approved by the school. In addition, online parenting forums, parenting associations and groups will be approached to promote the study. Advertisements will also be placed on community locations frequented by parents such as public libraries and community health centres. Parents will self-select by registering themselves and one child for the study online.

#### Inclusion criteria

Parent participants: Parents or guardians of at least one child aged 8 to 11 years (inclusive), who reside in Australia, are fluent in English, have regular access to the Internet and an email account are eligible for the study. Parents are able to participate even if their child opts out. Child participants: children of participating parents/guardians, aged 8 to 11 (inclusive), who reside in Australia, are fluent in English, will be included in the study. The child’s parent or guardian must have provided consent for their child’s participation, and the child must provide informed verbal assent to participate. Only one parent and one child per family are able to participate in the trial.

There are no exclusion criteria in relation to the child participant’s mental health status. While the Guidelines and the intervention are targeted at parents of children aged 5–11 years, the age range of the study sample is restricted to children aged 8–11 years in part due to the availability of psychometrically sound child-report measures that also have parent-informant reports; and in part, to limit the heterogeneity of the sample given the vast developmental differences between children aged 5 and children aged 11.

### Procedure

A flowchart outlining the study procedures is shown in Fig. 1. Interested parents will be directed to the trial website to review the Explanatory Statements (Parent and Child). Specifically, participants will be informed of the following: (1) study inclusion and exclusion criteria; (2) the two arms of the trial and their chance of being assigned to one of the arms; (3) the potential risks and benefits of participation; (4) privacy and confidentiality arrangements; (5) that the check-in calls provide them with an opportunity to ask any questions related to the study procedures, but will not provide any therapeutic support; and (6) contact helplines should they require immediate professional help. Parents who would like to proceed to participate in the study with their child will be asked to submit their informed consent and contact details on the trial website. One of the project managers (LMNF or WHS) will first screen registrations for eligibility. Subsequently, a member of the research team will contact the child participant at a pre-arranged time to obtain verbal assent, and then guide him or her through the online assessment over the phone as required. To assist child participants with literacy issues, the website provides audio clips which dictate the assessment instructions and items to participants when clicked.

Following submission of the child baseline assessment, an automated email will be sent to the parent containing the link to their online baseline assessment. Upon completing their baseline assessment, parents allocated to the intervention group will receive their tailored feedback report, a copy of the Guidelines, and the online parenting program comprising the recommended modules (out of a possible 12). Parents allocated to the control group will receive the first of their eight weekly factsheets.

Parents in the intervention group will receive a 5-min, weekly phone call from a researcher, starting 7 days after completing their baseline assessment and receiving their personalised feedback report, and every week thereafter for at least 8 weeks. The total number of calls will match the total number of modules selected plus one, with the first call focusing on the feedback report and supporting parents’ familiarisation with their online modules. Research staff will be trained to make these calls following a standard script and will not provide individual advice or therapy. The aims of these calls are to enhance parental engagement and ensure that parents progress smoothly through their allocated program until completion. Parents in the control group will receive a 5-min, weekly phone call from a researcher, starting 7 days after completing their baseline scale, and every week thereafter for 8 weeks to match the number of factsheets received.

At post-intervention (3-month) and each of the follow-up assessments (12-month and 24-month), children and parents will complete the same set of measures using the same procedure as the baseline assessment. Participants can opt out at any stage of the study. Verbal assent will be obtained from the child prior to asking them to complete each follow-up assessment.

#### Randomisation and blinding

Immediately after parents submit their baseline assessment online, the trial website is programmed to run a stratified random allocation sequence (based on parent gender) to allocate parent-child dyads to either the control or intervention group.

As such, allocation is concealed from both participants and researchers prior to assignment and baseline assessment. The ‘people administering the treatment’ (for both intervention and control groups) are also masked from the assignment as the intervention is delivered online by an automated trial website. Child informants of the outcomes of interest are assumed to be blinded because they are not direct recipients of the intervention (i.e. parenting program).

### Interventions

#### Intervention group

The ‘Parenting Resilient Kids’ intervention comprises:

1. An individually-tailored feedback report that highlights areas of strength in parenting (i.e., concordant with the Guidelines) and areas for improvement (i.e., not concordant with the Guidelines),
2. An interactive online parenting intervention (up to 12 modules) to support parents in making changes to the identified areas for improvement. Module recommendations are based on responses to the baseline parenting scale.

Feedback reports will be available online immediately after the baseline assessment is completed. Parents will also be emailed a copy of their feedback report and the Guidelines. Five days later, parents will be emailed a link to access their individually-tailored interactive parenting program. Parents can further tailor their parenting program by deselecting recommended modules and/or selecting additional modules. Parents then confirm their selection and can commence their personalised program. The 12 modules comprising the intervention are derived from topics covered in the Guidelines. Modules include illustrations, vignettes, interactive activities, goal-setting exercises, and an end-of-module quiz with immediate feedback to consolidate learning. Module topics and rationale for inclusion are shown in Table 1.

The website is programmed to unlock one module per week from each parent’s program, until all modules in their personalised program have been unlocked irrespective of whether parent has completed preceding modules. Each module will take 15–25 min to complete. Once parents have completed all the modules in their personalised program, they have ongoing access to all 12 modules for the duration of the RCT (up to 4 years).

#### Control group

Parents in the control group are provided with a standardised package of educational materials about child development and wellbeing via the trial website (see Table 2). Parent participants receive an email each week, which provides a link to the corresponding webpage with their information factsheet for that week, for 8 weeks (to match the expected mean number of modules received by the intervention group). This package provides general information to parents as opposed to tailored, actionable strategies, and is designed to represent a selection of resources that are available to parents as part of the current health promotion approach for child wellbeing. The materials are adapted from credible existing resources provided on the Raising Children Network website (www.raisingchildren.net.au).

**Table 2:** Factsheet topics and order of presentation

Order of presentation	Topic	Synopsis
Factsheet 1	Child development in the pre-teen years: an overview	Helps parents understand their child’s physical, emotional and social changes in the pre-teen years.
Factsheet 2	Behavioural changes in the pre-teen years	Helps parents understand common behavioural concerns in the pre-teen years and learn ways to encourage good behaviour
Factsheet 3	Connecting and communicating	Helps parents understand the importance of staying connected with their child and provide ideas on ways to improve communication with their child
Factsheet 4	Internet safety	Provides parents with ideas on how to help their child use the Internet safely and responsibly
Factsheet 5	Health and wellbeing in the pre-teen years	Helps parents understand the importance for their child to have healthy lifestyle habits
Factsheet 6	Nutrition during the pre-teen years	Provides parents with ideas on making healthy food choices for their child
Factsheet 7	School and education in the pre-teen years	Helps parent understand how they can support their child’s learning and education
Factsheet 8	Building a strong positive relationship with the school	Provides parent with ideas on how to be involved in their child’s school and to build a positive relationship with the school

### Outcome measures

The following primary outcomes were measured: changes in parental concordance with the Guidelines and changes in child anxiety and depressive symptoms. The secondary outcomes measured were child report of parenting, parent report of general family functioning, and child and parent health-related quality of life.

#### Primary outcome measures

Change in parental concordance with the Guidelines ‘How to reduce your child’s risk of depression and clinical anxiety: strategies for parents of primary school-aged children’ will be assessed by a parenting scale developed for use in this study. The scale consists of 83 questions, and is a criterion-referenced measure assessing parents’ current parenting practices (key parenting risk and protective factors) against specific recommendations in the Guidelines.

The 25-item Revised Children’s Anxiety and Depression Scale (RCADS) [24] will be administered to both parents (parent-report form) and child (child-report form) participants aged 8 to 11 inclusive. Change in child anxiety symptoms, as measured by the 15-item Total Anxiety subscale score, and child depressive symptoms, as measured by the 10-item Depression subscale score, will be tracked.

#### Secondary outcome measures

Child’s report of parenting is assessed by the 10-item Acceptance/Rejection subscale in the latest revision of Schaefer’s Children’s Report of Parent Behaviour Inventory (CRPBI-30, [25] and the 8-item Psychological Control Scale-Youth Self-Report (PCS-YSR) [26]. Each of these scales assesses some aspects of parenting covered in the parenting scale and will be used as a complementary measure for assessing parenting, as well as to form part of the validation of the newly developed parenting scale. This measure is completed only by child participants in Grades 5 and 6 (aged approximately 10–11), to ensure that children are able to provide valid reports on these measures. On these parenting measures, child participants are asked to report on their participating parent only. Parent participants will also complete the parent versions of the CRPBI and PCS-YSR.

General family functioning will be measured by parents’ reports on the 12-item General Functioning subscale of the McMaster Family Assessment Device [27]. Parents’ health-related quality of life will be measured by the 35-item Assessment of Quality of Life (AQoL-8D). The dimensions included in AQoL-8D are: independent living, pain, senses, mental health, happiness, coping, relationships and self-worth [28].

Child’s health-related quality of Life will be measured by Child Health Utility 9D (CHU-9D) and KIDSCREEN-27. The 9-item CHU-9D [29] assesses children’s self-report functioning across nine domains (worry, sadness, pain, tiredness, annoyance, school, sleep, daily routine and activities). The KIDSCREEN-27 [30] developed for children 8–18 years old, and has both self-report and parent proxy versions. It contains 27 items in five domains: physical well-being (five items), psychological well-being (seven items), autonomy and parents (seven items), peers and social support (four items), and school environment (four items). The CHU-9D will be completed by the child participant.

As part of program evaluation, parents in both groups will also be asked six to eight questions relating to the program they have received at each of the follow-up assessments.

#### Protocols for adverse events

Children who express distress during any of the child assessment calls will be contacted by either one of the project managers (LMNF or WHS) or the chief investigator (MBHY), all of whom are either provisional or registered psychologists. They will assess the level of acute risk and refer parent and child to appropriate follow-up options as required. Parents are provided contact details for the researchers and are encouraged to communicate any concerns about their participation in the program. A T-score of 70 or more on the Revised Children’s Anxiety and Depression Scale (RCADS) is used in the current study for detecting elevated child symptoms [24]. If both child and parent report elevated child symptoms, the project managers (LMNF and WHS) will follow-up with the parent participants, to encourage them to discuss these concerns with their child, as well as to provide information about the services they may wish to use if required. Participants are free to withdraw from the research or request that their data be withdrawn at any time.

#### Training for fidelity

Research staff are trained to conduct the weekly check-in calls with parents using a standard script (i.e. standard list of questions and prompts). At least one project manager (LMNF or WHS) will oversee the daily operation of the trial, and two research assistants will conduct the phone calls to parent and child participants. Extensive efforts will be made to minimise non-response and drop-out rates, including reminder calls, text messages, emails, participant reimbursement, and child birthday cards.

#### Confidentiality

Information obtained from the research will only be accessible by the researchers, and all electronic files will be password protected. Reports arising from the research will only include group data, and individual participants will not be identifiable in any way. As discussed in the adverse events protocol above, if both child and parent reported anxiety or depression scores on the RCADS are elevated, only the participating parent will be informed via email. However, no individual data will be provided to the parent. All data will be securely destroyed after a minimum of 5 years from when the final report of the study is published.

### Statistical analysis

The primary analyses will be intention-to-treat analyses. To assess group differences in changes in primary and secondary outcomes across time points, a series of mixed models repeated measures (MMRM) will be conducted. Little’s missing completely at random (MCAR) test will be used to analyse the extent and pattern of missing data.

## Discussion

Previous research suggests that parents can play a critical role in the prevention of anxiety and depressive disorders in their children. The Parenting Resilient Kids Program was developed as a web-based parenting intervention for parents of children aged 5–11 and was designed to be practical and parent-friendly, yet firmly rooted in research evidence. This RCT will examine whether compared to providing parents with a standardised package of educational factsheets, the Parenting Resilient Kids program leads to changes in parenting factors that are important in preventing child depression and anxiety in the short term (3 months after baseline), and child depressive and anxiety symptoms in the medium term (12 months post-intervention) and longer term (24 months post intervention). The RCT will also examine whether, compared to the control group, parents who have received the Parenting Resilient Kids intervention would show significant changes in children’s and parents’ health-related quality of life. If the Parenting Resilient Kids intervention is found to be effective, it can then be disseminated widely as a cost-effective preventative parenting program leading to better mental health and quality of life outcomes for primary school-aged children. This carries the potential to alter the developmental trajectory of anxiety and depressive disorders across the lifespan. Findings from this study may also contribute to the knowledge base for designing effective web-based prevention programs for parents and the extent to which parents can be engaged and motivated to change through a minimally guided parenting program.

## Trial status

This trial is currently recruiting participants till end of March 2018. A total of 1894 primary schools were contacted across Australia, of which 358 agreed to advertise the study. To date, the study has recruited 321 parent-child dyads, which is 94% of the required sample. The first participant was enrolled on 8 March 2017.

## Additional file

Additional file 1: SPIRIT checklist. (DOCX 27 kb)

## Abbreviations

ANZCTR: Australian New Zealand Clinical Trials Registry
AQoL-8D: Assessment of Quality of Life
CRPBI-30: Children's Report of Parent Behaviour Inventory
DET: Department of Education and Training
MCAR: Missing completely at random
MMRM: Mixed Models Repeated Measures
PaRK: Parenting Resilient Kids
PCS-YSR: Psychological Control Scale-Youth Self-Report
RCADS: Revised Children’s Anxiety and Depression Scale
RCT: Randomised controlled trial
